# Heterochronic changes in gene expression underlie placental evolution in the fish family Poeciliidae

**DOI:** 10.1098/rspb.2025.0573

**Published:** 2025-07-16

**Authors:** Jing Li, Polly Campbell, Axel Meyer, David Reznick

**Affiliations:** ^1^ Biology, University of Konstanz Department of Biology, Konstanz, Baden-Württemberg, Germany; ^2^ Department of Algal Development and Evolution, Max Planck Institute for Biology Tübingen, Tubingen, Baden-Württemberg, Germany; ^3^ Department of Biology, Oxford University, Oxford, UK; ^4^ Evolution, Ecology, and Organismal Biology, University of California Riverside, Riverside, CA, USA

**Keywords:** evolution of complexity, convergent evolution, placenta, transcriptomics, Poeciliidae, live-bearing fish

## Abstract

Placentas evolved nine times in the fish family Poeciliidae. Each time, the egg follicle is the maternal contribution to the placenta. In non-placental species, the follicle fully provisions the egg before fertilization. In placental species, provisioning continues throughout development, and the follicle becomes a more elaborate, well-vascularized organ. We generated transcriptomes for follicles from yolking eggs and developing embryos from two pairs of closely related placental and non-placental species that represent independent origins of placentation plus one non-placental outgroup. We identified genes expressed in eggs but not embryos of non-placental species that continue to be expressed during embryonic development in placental species. Their functions include the maternal transfer of nutrients and immunity. We then reconstructed the ancestral state of the non-placental common ancestor of each species pair and identified genes that were either upregulated or downregulated in developing embryos of placental species relative to non-placental species. These include clusters associated with lipid metabolism, immune response and tissue structure. The two placental lineages were convergent in the function of these genes, but few genes were in common between them. Thus, diverse gene regulatory changes converge on shared essential functions in the independent origins of a complex trait.

## 1. Introduction

Understanding the evolution of complex traits poses a special challenge because most manifestations of complexity are only represented in extant organisms as intricate end products that reveal little about the transition between their origin and present state. Because most such organs rarely fossilize, their origin is lost to history. Success in characterizing how complex traits evolve is facilitated in those rare cases that, by chance, capture some of this history in living organisms. For example, Recknagel *et al.* [1] gained insights into the genetic basis of the evolution of live-bearing in a lizard (*Lacerta vivipara*) because some populations still lay eggs. There is hybridization between live-bearing and egg-laying individuals, thus creating the natural equivalent of a quantitative trait locus (QTL) experiment.

The mammalian placenta is an example of what appears to be an inaccessible transition between an ancestral state and present-day complexity. It arose more than 180 million years ago in the common ancestor of marsupial and eutherian mammals [2] and is an essential feature of prenatal development in all extant species. In eutherian mammals, the placenta’s roles in maternal provisioning and maternal–fetal gas exchange are augmented by endocrine and immunoregulatory functions that maintain pregnancy and mediate maternal immune response to implantation and prolonged exposure to fetal antigens [2]. While some aspects of placental evolution can be inferred from the genomes of living organisms [3–5], most of what we learn from the study of contemporary mammals is how the placenta diversified within the Mammalia (e.g. [6,7]) rather than how and why it originated. However, the equivalent of the mammalian placenta evolved in other lineages. Some provide favourable material for characterizing how transitions to complexity were accomplished.

Placentas have evolved multiple times in the fish family Poeciliidae [8]. The family includes three clusters of close relatives with or without placentas [9,10]. Ancestral state reconstructions reveal that the common ancestor of the family likely lacked a placenta and that the placenta evolved at least nine times within the family [11]. This combination of multiple, independent origins with clusters of closely related species with and without placentas creates the potential to address how and why the placenta evolved in ways that are not possible for mammals.

Anatomical studies of the Poeciliid placenta reveal that the follicle, which is an envelope of maternal tissue that provisions eggs in all vertebrates, is always the maternal contribution to the placenta. In mammals, the follicle erupts, releasing the egg into the oviduct. In the Poeciliidae, the embryo develops inside the follicle. The rupture of the follicle occurs at birth. Placental species have enhanced follicular blood circulation and thickened folds of tissue and/or dense microvilli on their inner surface that are likely adaptations for the enhanced delivery of nutrients to the developing embryo [12–15].

### (a) The neoteny–hypermorphosis hypothesis

The follicle is thus the focus of maternal adaptations to placental reproduction throughout the family. Because it is not physically integrated with embryonic tissue, it is easy to isolate and hence provides a promising substrate for studies of placental evolution. While the placenta, by definition, is an integration of maternal and embryonic tissue, fate has not been so kind in defining an equivalent, easily isolated tissue that represents the embryonic contribution to placentation, so we begin with the follicle.

We propose the use of two concepts associated with heterochrony for interpreting how follicular evolution contributes to the evolution of placentation. The classic application of heterochrony involves the description of adults of descendant species as comprising a combination of juvenile and adult characters of the ancestor. For example, the axolotl (*Ambystoma mexicanum*) foregoes metamorphosis and becomes sexually mature while retaining larval morphology. Here we apply this concept to the follicle, rather than to the whole organism.

Follicular evolution involves two types of heterochrony. In non-placental species, the follicle fully provisions the egg before fertilization and then is reduced to a diaphanous envelope. In placental species, the follicle retains its provisioning capacity so that it can support the growth of developing offspring. This retention of provisioning that occurs only in the egg of the ancestor can be described as neoteny, or the retention of a trait in a descendant species to a more advanced stage of development than seen in the ancestor.

The second type of heterochrony that applies to follicular evolution is hypermorphosis. De Beer [16] observed that ‘…if the time when development stops is *relatively* delayed, it will be possible for the descendant to add characters on to the adult ancestral stage (p. 65)’. The follicle of placental species continues to develop after the egg is fertilized and embryogenesis begins. As part of this extended development, it acquires characters not seen in the follicles of lecithotrophic ancestors. The follicle becomes highly vascularized and thickened, with the inner surface lined with microvilli. This extended development of the follicle to a stage beyond what we see in the non-placental ancestor thus represents hypermorphosis. The maternal contribution to the evolution of the placenta is therefore potentially the product of two types of heterochrony—neoteny and hypermorphosis.

We use this interpretation of the evolution of the follicle to identify genes that contribute to each type of heterochrony and hence are promising candidates for future study. Genes expressed only in yolking eggs of non-placental species but that retain expression at later stages of embryogenesis in placental species are neoteny candidates. Hypermorphosis candidates are either newly recruited genes that are normally expressed elsewhere in the embryo or adult or are genes that are normally expressed in the follicle of the non-placental ancestor but suppressed in placental species.

We sequenced follicle transcriptomes from yolking eggs and developing embryos of two pairs of closely related species, one placental and one non-placental, which represent two independent origins of placentas. We do the same for a non-placental outgroup. As done by Lynch *et al.* [3], we perform an ancestral state reconstruction of gene expression in the common ancestor of each pair of closely related placental and non-placental species. Doing so enables us to focus on differences in gene expression that evolved during the descent of each pair of species from a common ancestor that lacked a placenta.

Vitellogenin receptors are an example of a potential component of neoteny because they play a role in provisioning the developing eggs of egg layers and, presumably, non-placental live bearers. Vitellogenins are dimeric proteins produced by the liver, then incorporated into the egg via receptor-mediated endocytosis [17]. These proteins are like a smorgasbord, with phosphate, lipid and carbohydrate components, plus are carriers of important ions (e.g. calcium, zinc and iron), minerals and some vitamins. Once within the egg, they are processed into their separate components and incorporated into the yolk. The evolution of placentation could include retention of these functions during embryogenesis. Retained expression of vitellogen receptors in the follicles of developing embryos would be one indicator of such neoteny. In fact, maternal provisioning of vitellogenin persists throughout embryonic development in three species of matrotrophic Goodeidae [18,19]. Candidate genes associated with hypermorphosis might be those associated with angiogenesis (development of the circulatory system), the production and transfer of nutrients and the suppression of the maternal immune response to increased exposure to foreign antigens.

The two paired comparisons are representatives of the southern (*Poeciliopsis gracilis*, non-placental; *Poeciliopsis presidionis*, placental) and northern (*Poeciliopsis infans*, non-placental; *Poeciliopsis prolifica*, placental) clades of the genus *Poeciliopsis. Poeciliopsis presidionis* has a thick-walled follicle with dense microvilli on the inner surface and a relatively thin-walled yolk sac, whereas *P. prolifica* has a thinner, smoother follicle and a proportionally thicker, more highly vascularized yolk sac [14]. Their thinner, relatively smooth follicles are still distinctly thicker and more highly vascularized than those of non-placental species [14]. The time to common ancestry for each of our focal placental species and their closest non-placental relative is 0.75 myr for *P. prolifica* and 2.36 myr for *P. presidionis* [9]. Our non-placental outgroup is *Micropoecilia picta*.

Panhuis *et al.* [20] generated descriptions of gene expression in the follicles of different-stage embryos of *P. presidionis* and *Poeciliopsis turneri*, sister species that inherited the placenta from a common ancestor. Guernsey *et al.* [21] compared the transcriptomes of follicles from advanced embryos of *Poeciliopsis retropinna* (placental) versus *Poeciliopsis turrubarensis* (non-placental). *Poeciliopsis retropinna* belongs to a clade that represents a third independent origin of placentation in the genus *Poeciliopsis. Poeciliopsis turrubarensis* is in the southern clade, along with *P. gracilis, P. presidionis* and *P. turneri*. Both studies found that genes associated with nutrient transport and biosynthetic processes were expressed in the follicles of placental species. The current study builds on this earlier work by performing analyses of gene expression at four stages of embryonic development in two paired comparisons of closely related placental and non-placental species that represent two independent origins of placentas and explicitly testing for two patterns of heterochronic gene expression.

## 2. Methods

Our breeding populations of all five species were the source of the gravid females used in this study. The collection of data for each population is given in the electronic supplementary material, table S1. All research was executed as defined in the UC Riverside #110 approved protocol.

We quantify maternal provisioning with the ‘matrotrophy index’ (MI), which is the dry mass of embryos at birth divided by the dry mass of the egg at fertilization. These estimates are derived from regression lines fitted to stage of development (*x*-axis) by mean dry embryo mass (*y*-axis) data derived from the dissection of at least 15 females per collection. MI values of less than one mean that the embryos lose weight during development. The modal value for non-placental species is 0.7 [22]. Placental species instead have MI values greater than one because the embryos gain weight during development. The MI values for *P. infans* and *P. prolifica* are 0.86 [9] and 8 [23], respectively. The MI values for *P. gracilis* and *P. presidionis* are 0.69 and 21.5, respectively [9]. The MI value for *M. picta* is 0.64 [24].

**Figure 1.**
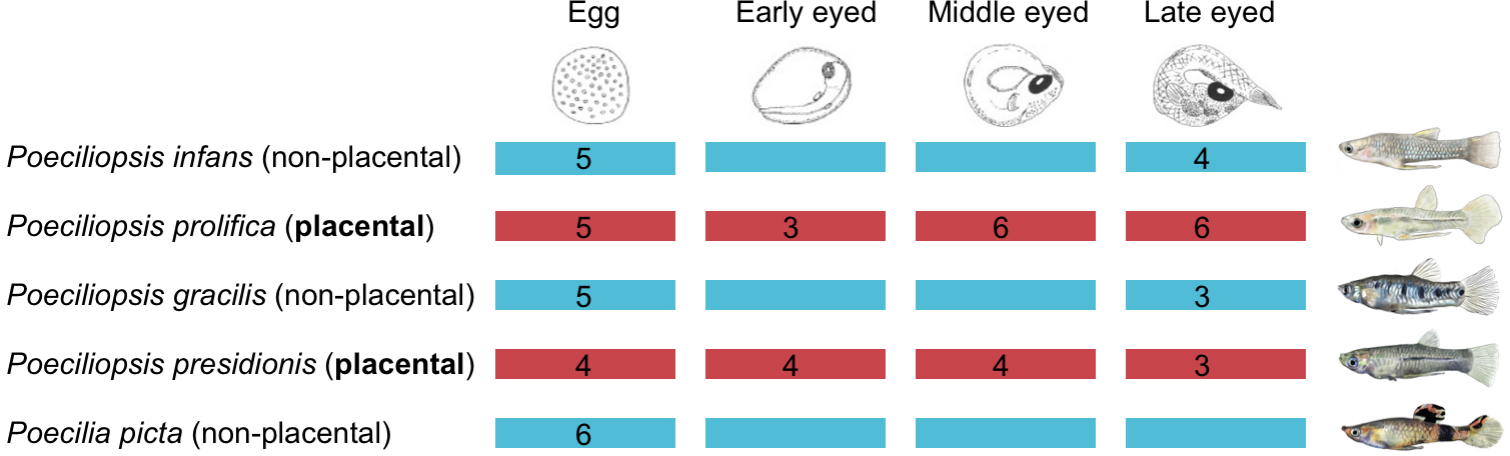
Sample collection. In total, 58 follicles from four continuous developmental stages of five species were collected and sequenced.

Gravid females were euthanized with an overdose of buffered tricaine methane sulfonate (MS-222). The ovary was removed, embryos isolated, and their stage of development defined as in [25]. Our sampling scheme, including the stages of development that were represented and the number of samples per stage, is summarized in figure 1. All species of *Poeciliopsis* have superfetation, which means that the female may be carrying multiple litters of developing young in distinctly different stages of development, enabling us to obtain follicles from more than one litter of offspring from individual females. All follicles were placed into RNAlater (Invitrogen), stabilized at 4°C overnight, then stored at −20°C.

### (a) RNA extraction and transcriptome sequencing

Total RNA from 58 follicle tissues was isolated using TRIzol reagent (Thermo Fisher Scientific) according to the supplier’s recommendation. Custom sequencing (BGI genomics) of TruSeq libraries generated 30−35 million 150 bp paired-end clean reads for each sample on the BGISEQ platform.

### (b) Genome annotation

The reference genomes for all five species were downloaded from NCBI (accession numbers are listed in the electronic supplementary material, table S2) and annotated using a pipeline adapted from Funannotate [26]. In brief, the repeats of the genome were predicted using standard RepeatMasker (v. 4.1.2) [27] procedures, with the default transposable element Dfam (v. 3.3) database [28] and a de novo repeat library constructed using RepeatModeler (v. 2.0.2) [29] with default parameters. Protein-coding genes were annotated by collecting and synthesizing gene evidence from homology, transcriptome and *ab initio* predictions with EVidenceModeler [30]. We blasted annotated genes against the InterProscan, Swiss-Prot and RefSeq databases, identified protein domains and assigned gene symbols and names.

### (c) Identification of one-to-one orthologues

We identified one-to-one orthologues between *P. prolifica,* for which there is the highest quality genome among our study species [31], and other species by considering both reciprocal best BLASTP hits and synteny. First, we conducted BLASTP for all protein sequences from *P. prolifica* and other species with an *E*-value cut-off of 1 × 10^−7^. We then identified reciprocal best hit (RBH) orthologues between *P. prolifica* and every other species based on alignment score, alignment rate and identity. From these RBH orthologues, we retained those pairs with conserved synteny across species. Synteny was determined based on flanking genes: if the RBH orthologous gene pair shared the same flanking genes, we kept them for the downstream analyses. Finally, we merged the pairwise orthologue list according to the *P. prolifica* genome coordinates. In this way, we produced a final list of 14 663 one-to-one orthologues across five species.

### (d) Gene expression profiling

We mapped the sequenced reads to each genome using HISAT2 [32] with default parameters. Only unique mapped RNA-seq reads were used in the fragments per kilobase of transcript per million mapped reads (FPKM) calculation. We used DESeq2 [33] to normalize FPKM across samples within species and generated an expression matrix for each species.

Principal component analysis (PCA) of normalized expression in follicle tissues based on the 14 663 one-to-one orthologues was performed in R using the prcomp function. In the PCA based on gene expression levels in FPKMs, species clusters overlapped, and separation between placental and non-placental species was weak. To reduce the noise caused by the continuous variation in FPKM values, we followed Mika *et al.* [34] and transformed quantitative gene expression values in FPKM into binary encoding—genes with FPKM ≥2.0 were coded as expressed (state = 1), genes with FPKM <2.0 were coded as not expressed (state = 0) and genes without data in one or more species were coded as missing.

### (e) Phylogenetic analyses

We used the binary-encoded follicle transcriptome dataset for phylogenetic analyses to reconstruct ancestral gene expression states [34]. Gene expression phylogenies from both egg and late stages were inferred with IQ-TREE2 [35] using the best-fitting model of character evolution determined by ModelFinder [36]. The best-fitting models were inferred to be GTR2 + FO + I + G4 (AICc = 170232.884) for egg stage and GTR2 + FO + I + I + R2 (AICc = 157608.139) for late stage. Node support was evaluated with UFBoot2 [26].

The ancestral transcriptome reconstruction was also conducted in IQ-TREE2 with parameters --ancestral --asr-min 0.50 and with the species phylogeny as input. The genes with reconstructed ancestral gene expression states were used for downstream analysis.

### (f) Identification of the neoteny candidate genes

We define neoteny candidate genes as ones that are expressed in the follicles of yolking eggs but not in the follicles of developing embryos of a non-placental species (i.e. *P. gracilis* or *P. infans*) but continue to be expressed in the follicles of the embryos of closely related placental species. We assume that genes associated with the transfer of nutrients from the mother to the yolking eggs of non-placental species will be active only while the eggs are being provisioned. Some of these same genes may remain active in the follicles of the developing embryos of placental species because these embryos continue to be provisioned throughout development. Based on these assumptions, we required that candidate neoteny genes (i) have at least fourfold higher expression in the late-stage follicles of the placental relative to the non-placental species, (ii) be upregulated by at least twofold in the egg stage relative to late stage of each non-placental species, and (iii) in placental species, have an absolute FPKM value of at least 2 for late-stage follicles and at least 1 for egg stages.

### (g) Identification of hypermorphosis candidate genes

Hypermorphosis can involve either the upregulation or downregulation of genes in the placental lineage relative to its non-placental close relative. All genes that are differentially expressed in the late-stage follicles of closely related placental and non-placental species pairs are potential regulators of the follicular features that facilitate extended maternal provisioning in the placental species. However, processes unrelated to placental evolution will contribute to gene regulatory divergence between species, and some differences in expression will reflect regulatory evolution in the non-placental lineages. To minimize the contribution of these background sources of differential expression, we required that candidate hypermorphosis genes have (i) at least fourfold higher or lower expression in the late-stage follicles of the placental relative to the non-placental species, and (ii) derived expression relative the common ancestor of each species pair *in the same direction* as the expression difference between species (i.e. either upregulated relative to the non-placental species and the common ancestor or downregulated relative to the non-placental species and the common ancestor). We used ancestral state transcriptome reconstruction to identify genes with derived upregulated and derived downregulated expression patterns: genes with FPKM >2 in the late-stage embryos of the placental species and FPKM <2 in the egg stage of the common ancestor of the species pair and genes with FPKM <2 in the late-stage embryos of the placental species and FPKM >2 in the egg stage of the common ancestor of the species pair, respectively.

### (h) Gene network and enrichment analyses

To test for evidence of functional interactions within gene sets of interest, we constructed gene networks in String [37] using all active interaction data sources, a minimum required interaction score of 0.4 (medium confidence) and query genes only (first shell). Gene networks were visualized and edited in Cytoscape [38]. Tests for Gene Ontology (GO) term overrepresentation were run in Panther [39]. We initially used zebra fish (*Danio rerio*) genes as background genes, but found that less than 44% of genes of interest in *Poeciliopsis* were annotated in the Panther zebra fish database. In contrast, more than 96% of these genes were annotated in the mouse (*Mus musculus*) database. Although fewer terms were significantly enriched using zebra fish genes as a background, we found considerable overlap with those enriched using mouse genes, indicating that orthologues share functional annotations. We therefore focus on the gene network and enrichment results for which mouse genes were the background, but provide results for both species’ backgrounds in the electronic supplementary material.

## 3. Results and discussion

### (a) Distinct but broadly convergent gene expression in placental species’ late-stage follicles

**Figure 2.**
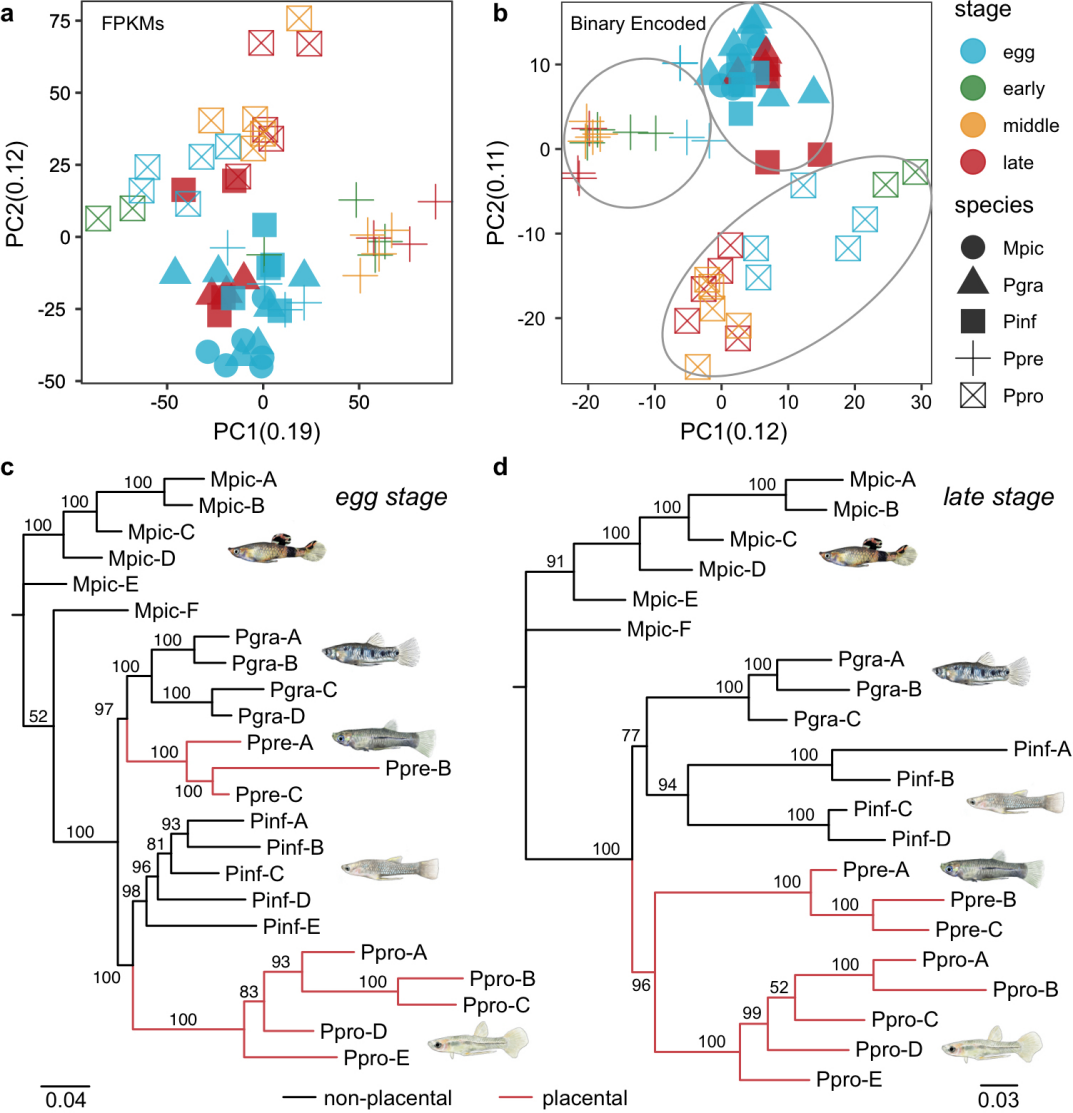
Clustering of follicle transcriptomes. (a,b) Binary encoding uncovers phylogenetic signal in transcriptome data. (a) PCA of gene expression levels (FPKMs) of follicle tissues does not group species and separate placental from non-placental species clearly. (b) Logistic PCA of the binary-encoded follicle transcriptome dataset clearly separates placental from non-placental species. ‘No’ = yolking eggs with no developing embryos present. ‘Early’ = eggs with early, uneyed embryos. ‘Middle’ = mid-eyed embryos, or those with eye pigment and the rudiments of fins. ‘Late’ = late eyed embryos, or those with developed fin rays. (c) Maximum-likelihood (ML) phylogeny of binary encoded follicle transcriptome data inferred by IQ-TREE2 under the GTR2 + FO + I + G4 model, which is consistent with the species phylogeny. All samples are from yolking egg stages. (d) ML phylogeny of binary-encoded follicle transcriptome data inferred by IQ-TREE2 under the GTR2 + FO + I + I + R2 model, which recovers placental and non-placental clades and does not reflect the species tree. All samples are from an advanced late embryonic stage. Fish icons were hand-drawn by Min Zhou from the University of Tübingen.

We first offer an overview of the comparative transcriptomics of the different stages of development. Our PCA was performed first on the raw dataset (figure 2a), then on binary coded data (figure 2b), for which a given gene was scored as either expressed (FPKM ≥2.0) or not expressed (FPKM <2.0). As observed by Mika *et al.* [34], we found that the binary-encoded data yielded cleaner discrimination among species, so we base our interpretation on that analysis. All developmental stages of all three non-placental species clustered tightly, implying similar patterns of gene expression. The yolking eggs (‘egg’ in figure 2a,b) and uneyed embryos (‘early’ in figure 2a,b) of the placental species, *P. prolifica* and *P. presidionis*, clustered close to the non-placentals, but the more advanced stages (‘middle’ and ‘late’ in figure 2a,b) became progressively more different from the non-placental species, indicating substantive changes in gene expression in the follicles derived from embryos of placental species. The trajectories of change of the two placental species diverged, indicating differences between the two in gene expression patterns during development. Because the samples were sequenced in two batches, we evaluated potential batch effects using PCA. The results show broad overlap between samples from the two batches and thus provide no evidence for an effect of batch on expression (electronic supplementary material, figure S1).

A phylogeny based on gene expression in eggs has the same topology as one obtained from DNA sequences of combined nuclear and mitochondrial genes (figure 2c; [8,9]). A phylogeny based on gene expression in follicles derived from advanced embryos instead grouped species by the mode of maternal provisioning (figure 2d). This reconfigured topology reflects the pattern of change in the function of the follicles associated with the advanced embryos of non-placental and placental species. In the non-placental species, the follicle likely facilitates oxygen exchange and perhaps waste disposal. In the placental species, the growth trajectory of the embryos is exponential [9], which means that the follicles must deliver ever-increasing quantities of resources. The fact that *P. prolifica* and *P. presidionis* group together indicates that the patterns of gene expression that unite these placental species to the exclusion of their closer relatives are greater than the differences between them seen in figure 2b [34].

### (b) Neoteny candidate genes are associated with metabolic and immune functions

**Figure 3.**
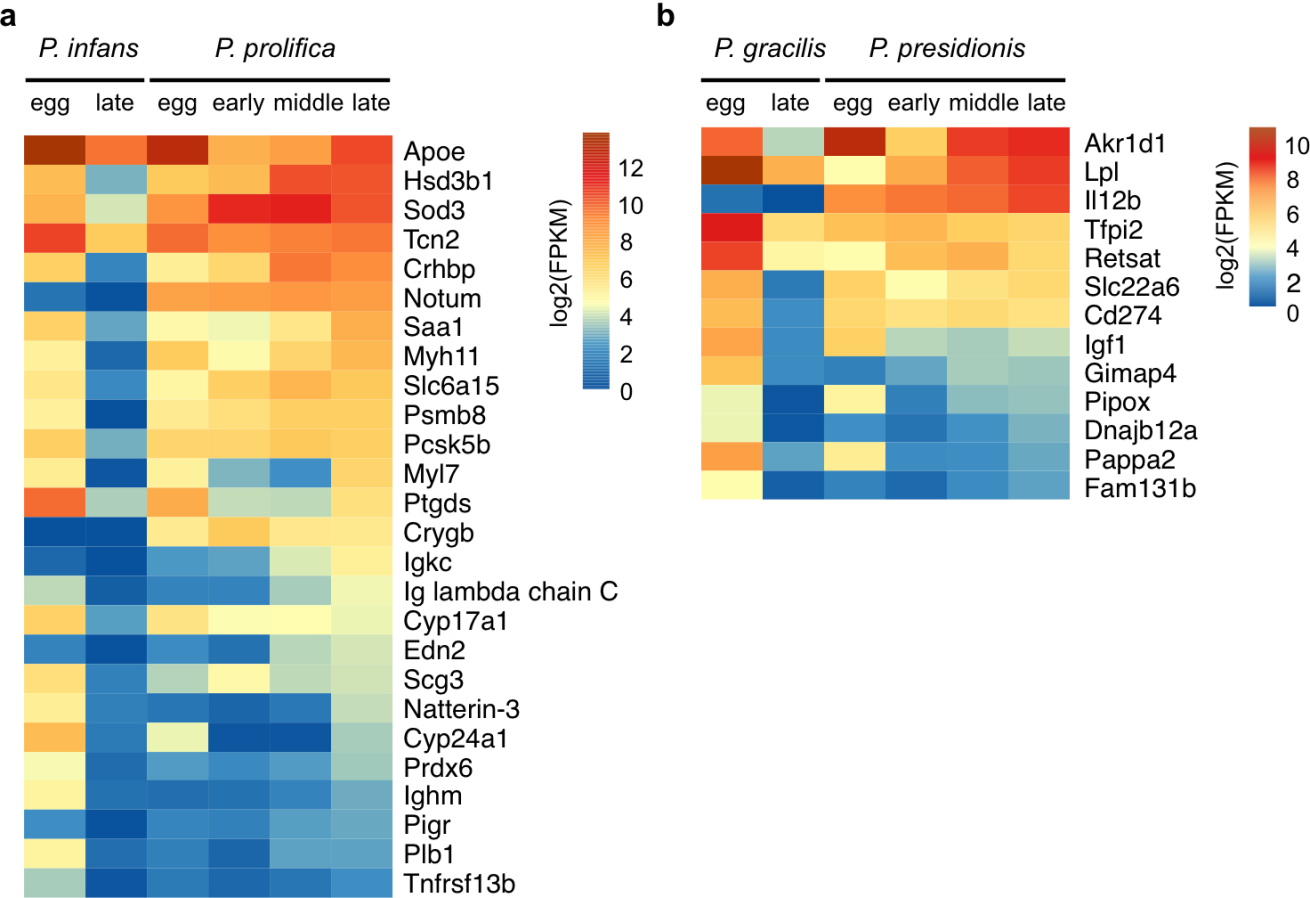
Test of the neoteny hypothesis. (a,b) Heat map illustrating the candidate neoteny genes in two placental and non-placental species pairs.

Relatively few genes met our neoteny criteria (figure 3). We found 26 candidate loci for the *P. infans–P. prolifica* species pair (figure 3a) and 13 candidates for the *P. gracilis–P. presidionis* species pair (figure 3b).

Neoteny candidate genes in *P. prolifica* were enriched for GO terms associated with lipid and steroid hormone metabolism and immunity (complete results in electronic supplementary material, file S1). Lipid metabolism genes (*Apoe, Hsd3b1, Saa1, Ptgds, Cyp17a1, Edn2, Cyp24A1, Prdx6* and *Plb1*) comprise 35% of neoteny candidates in *P. prolifica*. Neoteny candidate genes in *P. presidionis* were enriched for terms associated with the regulation of T cell proliferation and cytokine production (electronic supplementary material, file S1). While there were no genes in common between *P. prolifica* and *P. presidionis*, several genes in *P. presidionis* function in lipid metabolism (*Retsat, Lpl*), lipid transport (*Slc22a6*) and steroid hormone metabolism (*Akr1d1, Igf1*). Likewise, genes with immune system-related functions comprised 19–39% of each gene set (*P. prolifica: Psmb8, Igkc, Ighm, Pigr, Tnfrsf13b; P. presidionis: Il12b, Tfpi2, CD274, Igf1, Gimap4*).

While we did not see the predicted presence of vitellogenin receptors, the expression of genes involved in lipid metabolism and transport during embryonic development in both placental species is consistent with the expectation that genes associated with egg provisioning in non-placental species should continue to function in embryonic provisioning in their placental relatives. For example, *Apoe* in *P. prolifica* belongs to the vitellogenin-related apolipoprotein gene family [40,41], whose members facilitate transfer of maternal lipids to yolking eggs in oviparous species (e.g. teleost fish; [42]), and placental lipid metabolism in mammals [43]. Similarly, Lpl in *P. presidionis* is important to lipid deposition in the oocytes of non-placental teleosts [44–46] and hydrolyses maternal lipoproteins for transport across the placental barrier in mammals [47]. Several of the immune genes are involved in placental modulation of maternal immune response during mammalian pregnancy. For example, CD274 is an immunoinhibitor that is highly expressed in the mammalian trophoblast, where it suppresses maternal T cell attack of the fetus [48]. Interestingly, *CD274* is also upregulated in the female reproductive tract of the guppy (*P. reticulata*), a non-placental poeciliid, several hours post-insemination [49].

In conclusion, our expectation for the continued maternal provisioning of vitellogenin throughout embryonic development, as seen in three species of matrotrophic Goodeids [18,19], was not fulfilled. We nevertheless identified genes with potentially important roles in maternal nutrient transfer to the yolking eggs of non-placental species that remain active in follicles supporting developing embryos in placental species. The expression of immunity genes in the late-stage follicles of placental species is in line with the expectation that prolonged exposure to an allogenic embryo should require modulation of the maternal immune system.

### (c) Hypermorphosis candidate genes

**Figure 4.**
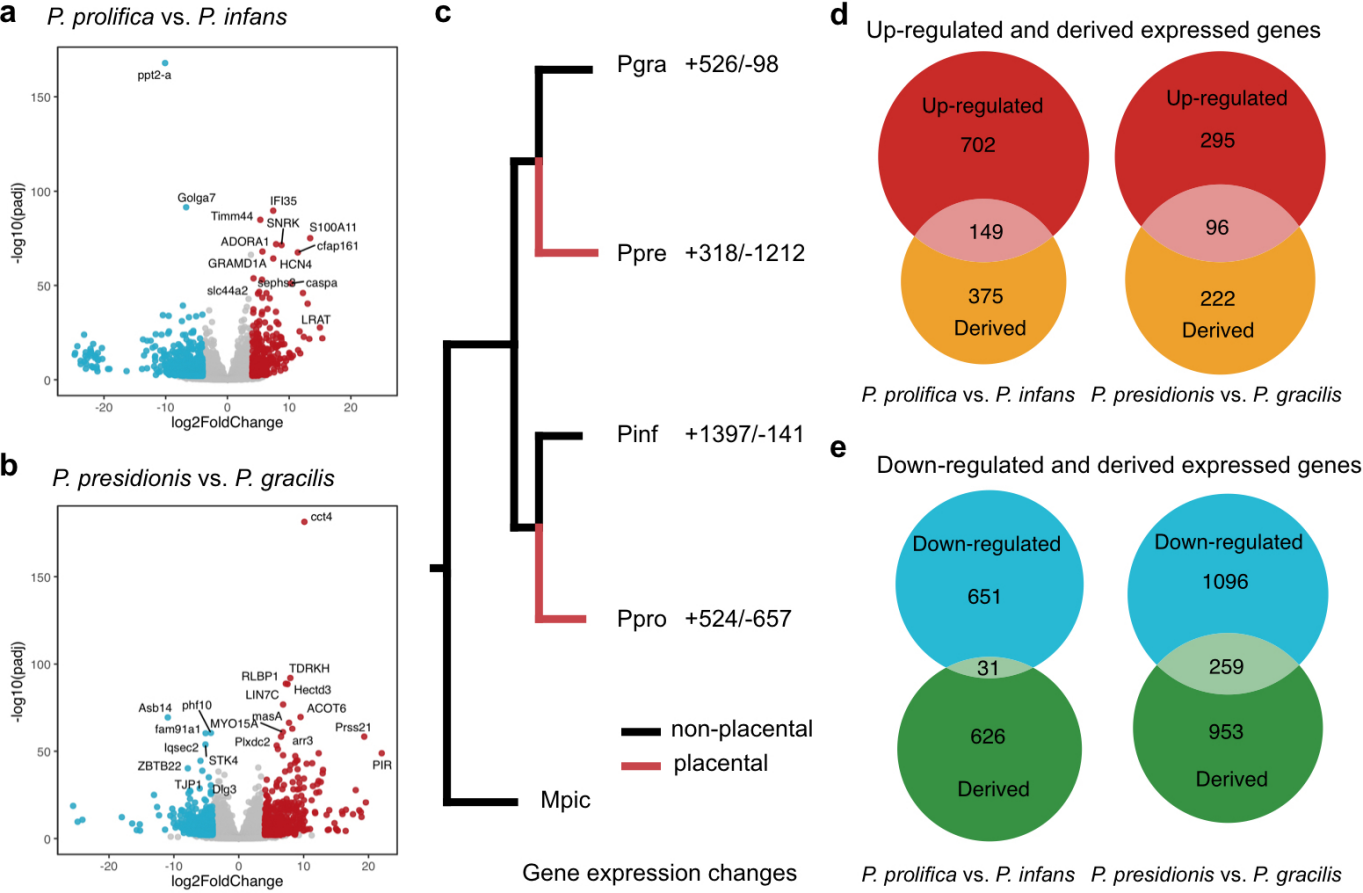
Test of the hypermorphosis hypothesis. (a,b) Differentially expressed genes in the late stage of follicle tissues between the two placenta and non-placenta species pairs. The blue dots show genes with higher expression in non-placental species, while red dots indicate genes with higher expression in placental species. (c) Ancestral expression reconstruction with species trees. Inset legend shows the number of gene expression changes from the root node to each species (+ = increased expression; − = decreased expression). (d) Venn diagrams with candidate upregulated hypermorphosis gene counts in the overlap between upregulated genes and genes with derived expression in the two placental species. (e) Venn diagrams with candidate downregulated hypermorphosis gene counts in the overlap between downregulated genes and genes with derived expression in the two placental species.

We first quantified differential gene expression in the follicles from advanced embryos of each pair of placental and non-placental species (figure 4a,b). In each case, red data points correspond to upregulated genes in the placental species, while blue data points correspond to downregulated genes in the placental species. There were fewer differentially expressed genes between *P. prolifica* and *P. infans* (1533 genes) than between *P. presidionis* and *P. gracilis* (1746 genes; figure 4d,e). Given that *P. prolifica* and *P. infans* are the younger species pair [6,42], this suggests that the recruitment of new genes associated with placental function is progressive. We then identified genes with derived expression patterns in the placental species of each pair. Here again, we found fewer genes with derived expression in *P. prolifica* (1181 genes) than in *P. presidionis* (1530 genes; figure 4d,e). Candidate hypermorphosis genes are in the union of these sets: genes that are either upregulated or downregulated in the placental species relative to both the non-placental close relative and the common ancestor of the species pair (hereafter, derived-up and derived-down genes; electronic supplementary material, file S2).

#### (i) Placental species recruit different genes to embryonic provisioning

**Figure 5.**
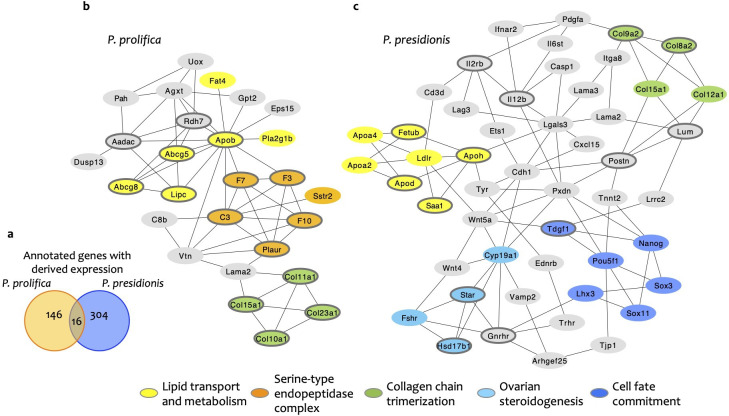
Functional interactions between genes with derived upregulated and downregulated expression in *P. prolifica* and *P. presidionis*. (a) Venn diagram showing low species overlap in the identity of genes with derived expression. (b) Gene network for *P. prolifica*, and (c) gene network for *P. presidionis*. Enriched functional clusters are indicated for lipid transport and metabolism (yellow), serine-type endopeptidase complex (orange), collagen chain trimerization (green), ovarian steroidogenesis (light blue) and cell fate commitment (dark blue). Networks constructed from the top 15 hub genes (outlined in dark grey) for each species. Full gene networks for both species are provided in the electronic supplementary material.

**Figure 6.**
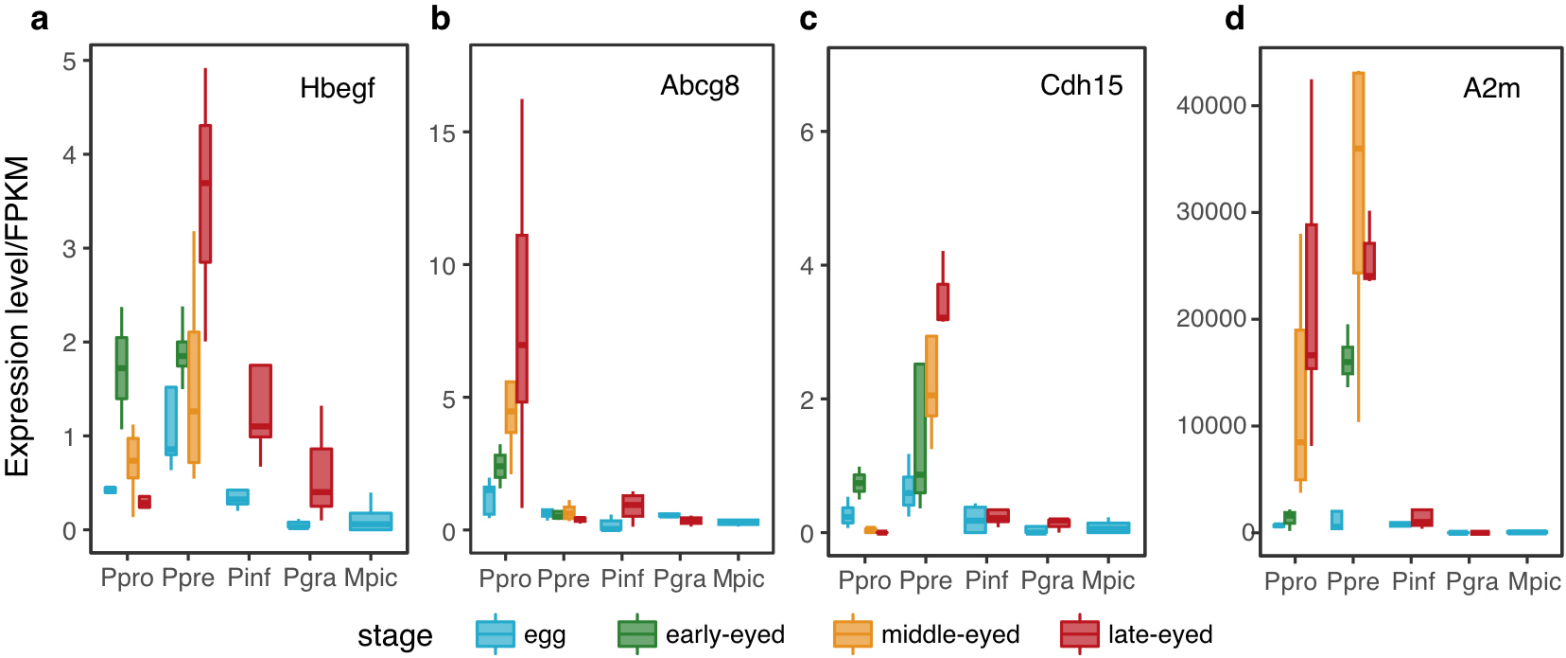
Stage-specific expression of representative hypermorphosis genes. (a*) Hbegf* (*P. presidionis*) is a uternine mediator of blastocyst implantation in mammals [56,57] . (b) *Abcg8* (*P. prolilfica*) and (c) *Cdh15* (*P. presidionis*) are imprinted in mammals. (d) *A2m*. ‘Stage’ represents the stage of development. ‘Egg’ = eggs with no developing embryos; ‘early eyed’ = neurula or embryos with unpigmented eye cups; ‘middle-eyed’ = embryos with pigmented eyes and fin rudiments; ‘late-eyed’ = embryos with body pigment and well-developed fins.

We found little evidence for convergent upregulation of the same genes in late-stage follicles of placental species. Of the 133 and 86 derived-up annotated genes in *P. prolifica* and *P. presidionis*, respectively, only four were common to both species (electronic supplementary material, file S2). There were close to an order of magnitude more genes with derived-down expression in *P. presidionis* (259 genes) relative to *P. prolifica* (31 genes), with 10 genes common to both species (electronic supplementary material, file S2). The total number of genes with derived expression in *P. prolifica* and *P. presidionis* is summarized in figure 5a. Representative derived-up genes are highlighted in figure 6a-c. Although the larger number of derived-down genes in the *P. presidionis/P. gracilis* species pair relative to *P. prolifica/P. infans* is consistent with longer time to common ancestry in the former, the magnitude of difference in gene numbers was unexpected. One possible interpretation is that the evolution of a thick-walled follicle supporting the development of the large *P. presidionis* offspring requires more extensive modification of gene regulatory pathways than does the evolution of the comparatively thinner follicle of *P. prolifica*.

Enriched GO terms for genes with derived expression were similarly species-specific. While *P. prolifica* genes were highly enriched for terms associated with cardiac development, together with activation of the complement system and regulation of intestinal lipid and cholesterol absorption, *P. presidionis* genes were enriched for multiple terms associated with metabolic processes, regulation of transport and reproductive system and brain development (electronic supplementary material, file S1).

#### (ii) Convergent recruitment of genes involved in lipid metabolism, tissue structure and immune response

In spite of minimal overlap in gene identity and associated enrichment terms, multiple genes with functions in lipid metabolism were recruited to late-stage follicles in both *P. prolifica* and *P. presidionis*. We therefore used network analysis to evaluate the potential for convergent recruitment of genes with parallel functions in extended maternal provisioning. Gene networks constructed from all annotated genes with derived expression in each species featured multiple species-specific clusters, three of which are common to both species: lipid absorption and metabolism, collagen chain trimerization and the complement system. Reduced gene networks comprised of hub genes (genes with the most functional connections to other genes in the network) and their closest interaction partners are shown in figure 5. Full gene networks are provided in electronic supplementary material, figures S2 and S3.

Convergent recruitment of functionally interconnected genes that mediate lipid transport (e.g. *Abcg5*, *Abcg8*, *Apob* in *P. prolifica; Apoa2, Apoa4, Apod, Apoh* in *P. presidionis*) and metabolism (e.g. *Lipc*, *Pla2g1b* in *P. prolifica; Fabp2, Ldlr* in *P. presidionis*) is consistent with the follicle’s function in provisioning embryonic growth and development in placental species. The fact that different members of these same functional classes of genes are prominent among the neoteny candidates suggests that transitions from static nutrient deposition in the egg yolks of non-placental poeciliids to dynamic nutrient transfer across development in placental species required the recruitment of additional genes to existing pathways. Collagen chain trimerization is essential to the structural stability of a wide range of tissues across Metazoa [50]. We speculate that the derived expression of multiple collagens in both placental species reflects an increase in follicular density and tissue complexity relative to non-placental species. Finally, the complement system is an effector of both innate and acquired immune responses in jawed vertebrates [51]. In mammals, balanced regulation of the complement system is essential to successful pregnancy [47]. Notably, complement system genes are expressed in both maternal and embryonic tissues at implantation [52], suggesting that embryonic self-protection from maternal immune response includes components of the complement system. Our evidence for the recruitment of complement system genes to the maternally derived placenta in both *P. prolifica* and *P. presidionis* motivates future investigation of the reciprocal activation of complement system genes on embryonic surfaces.

In *P. prolifica* only, complement system genes are functionally interconnected with a cluster of genes involved in a variety of immune-mediated responses to injury and infection that include the complement system (serine-type endopeptidase complex cluster; figures 5b and electronic supplementary material, figure S2). Other functional clusters unique to each placental species include genes involved in immune defence (T cell receptor complex cluster), the production of ovarian steroids and a suite of core developmental transcription factors in *P. presidionis* (ovarian steroidogenesis and cell fate commitment clusters; figures 5b and electronic supplementary material, figure S3) and regulators of cellular metabolism and energy balance in *P. prolifica* (AMP catabolism cluster; electronic supplementary material, figure S2).

Collectively, the results of this analysis suggest that the majority of genes with derived expression in late-stage follicles differ between the two independent origins of the placenta studied here in both identity and function. Against this background of dissimilarity, shared representation of genes involved in lipid transport and metabolism among candidate neoteny and hypermorphosis genes suggests that developmental extension and modification of these functions are of central importance to the evolution of maternal provisioning throughout embryogenesis. Similarly, the extended and derived expression of multiple immunity genes in both *P. prolifica* and *P. presidionis* points to an increase in maternal immune modulation in follicles exposed to fetal antigens.

#### (iii) Convergent expression of A2m

Two prior studies of follicular gene expression in placental and non-placental poeciliids identified *A2m* (alpha-2-macroglobulin) as highly expressed in the follicles of three placental species of *Poeciliopsis* but with little or no expression in the follicles of one non-placental species of *Poeciliopsis* [20,21]. Guernsy *et al.* [21] found that *A2m* was most strongly expressed on the surface of the microvilli that line the inner surface of the follicle of *P. retropinna*. In mammals, *A2m* regulates the remodelling of uterine vasculature during pregnancy [53,54] and suppresses the maternal immune response to developing embryos [55]. We found that *A2m* was expressed at low levels in the unfertilized eggs of our two placental species and in all assayed stages of development of our three non-placental species, but was very highly expressed in the embryo-containing follicles of the two placental species (figure 6d). If we add our results to the earlier papers, this means that *A2m* is expressed at high levels in follicles containing embryos in four placental species that represent three independent origins of placentation (*P. prolifica, P. presidionis, P. turneri* and *P. retropinna*) and expressed at much lower levels in the follicles of late-stage embryos from four non-placental species (*P. infans, P. gracilis, P. turrubarensis* and *M. picta*). This common pattern of association between *A2m* upregulation and the evolution of placentation clearly defines this gene as a promising target for further study.

*A2m* was not on our lists of candidate hypermorphosis genes because its expression level in the outgroup (*M. picta*) was above our cut-off for derived-up genes. However, the pattern of *A2m* expression is qualitatively similar to that of the four additional candidate hypermorphosis genes common to both placental species (electronic supplementary material, figure S4): upregulation in follicles supporting later stage embryos and uniformly low expression in all samples from non-placental species. Such expression patterns characterize all of the genes in the derived, upregulated category.

## 4. Conclusions

The repeated evolution of placental function in the follicles of live-bearing fish provides a rare opportunity to study the genetic underpinnings of a complex trait in replicates. Here, we use changes in follicular gene expression across development in two placental/non-placental species pairs to identify genes that are promising candidates for future study and to determine the extent to which convergence in function reflects convergent recruitment of the same genes or gene regulatory pathways. The patterns of expression in eggs and early embryonic stages are similar across all species, whereas expression in the two placental species diverges as development progresses (figure 2b). A phylogeny based upon gene expression in the yolk egg stages recovers relationships between placental/non-placental species pairs that are consistent with prior phylogenetic analyses (figure 2c; e.g. [8,9]). However, a phylogeny based on gene expression in follicles supporting late-stage embryos unites species by mode of maternal provisioning rather than evolutionary history (figure 2d). The placental species thus have enough in common to distinguish them from the non-placental species, while at the same time diverging from each other. We interpret the fact that the gene regulatory profile of each placental species is distinct, yet still more similar than to their non-placental relatives than to each other, as a measure of convergent evolution. Indeed, the extreme scarcity of specific candidate genes common to both placental species is a noteworthy feature of both neoteny (figure 3) and hypermorphosis (figure 4) candidate genes. Furthermore, while some of these genes play significant roles in mammalian placentation, most do not. We anticipated that a ‘path of least resistance’ for placental evolution would be the retention of mechanisms of egg provisioning in the non-placental ancestor for embryo provisioning in the placental descendant (neoteny). We found little evidence for such retention. Yet we did observe the retention or recruitment of genes in the same functional pathways, particularly among the hypermorphosis candidate genes (figure 5). We again interpret these outcomes as a measure of the independent origins of the placenta in each species, the polygenic nature of placentation and hence the diversity of options available to govern placental evolution. Another recent comparison of genes recruited during the evolution of placentation [58] revealed the same pattern of having few genes in common but broad overlap in function.

Griffith & Wagner [59] argue that the value of studying the evolution of the mammalian placenta lies in its recent origin relative to the other main organ systems. This recency facilitates the study of how such complexity evolves. The insights they gain from the mammalian placenta and the functionally equivalent organs that have evolved in many other organisms are that novelty arises from an integration of new genes and cell types plus old parts recycled for new functions via the recruitment of existing genes into new regulatory networks. For example, the amniotic membranes of mammals serve very similar functions to those of all amniotes, so their role in mammals just represents ‘…a shift in the physiological context in which these capabilities are employed’ [59, p. 2]. The recruitment of existing genes is a different manifestation of this recycling process. In placental mammals, the evolution of the placenta involved the recruitment of more than 1000 existing genes normally expressed in different tissues and/or at different stages of development. Here, the true novelty lies in the evolution of the regulatory elements that are responsible for this recruitment. Finally, novel placental genes have arisen via gene duplication or the domestication of retroelements.

The evolution of the poeciliid placenta follows this same pattern, with the modification of an existing tissue—the follicle—and the recruitment of new gene regulatory networks to support the associated change in follicle structure and extended provisioning of the embryo. Whether new genes and cell types also contribute to placental origins in Poecillidae remains to be determined. Indeed, some of the ‘hypermorphosis – derived-down’ candidate genes that we identified in placental Poeciliidae are ones that are upregulated and play key roles in mammalian placentas, which suggests some fundamental difference in underlying genetic control and perhaps also function. The virtue of the poeciliid placenta is that there have been at least nine independent origins, all much more recent than the mammalian placenta, sometimes with the retention of the ancestral state in close relatives. This diversity offers the promise of our being able to characterize the combined importance of contingency and fate in shaping the evolution of complexity.

## Data Availability

The RNA-seq data of all the samples have been deposited in NCBI under the Bioproject accession code PRJNA1129066. Supplementary material is available online [60].
